# Supplementary figures and images for: Curcumin induces mild anemia in a DSS-induced colitis mouse model maintained on an iron-sufficient diet
Source: PLoS One. 2019 Apr 26;14(4):e0208677. doi: 10.1371/journal.pone.0208677 (PMC6485613; doi:10.1371/journal.pone.0208677)

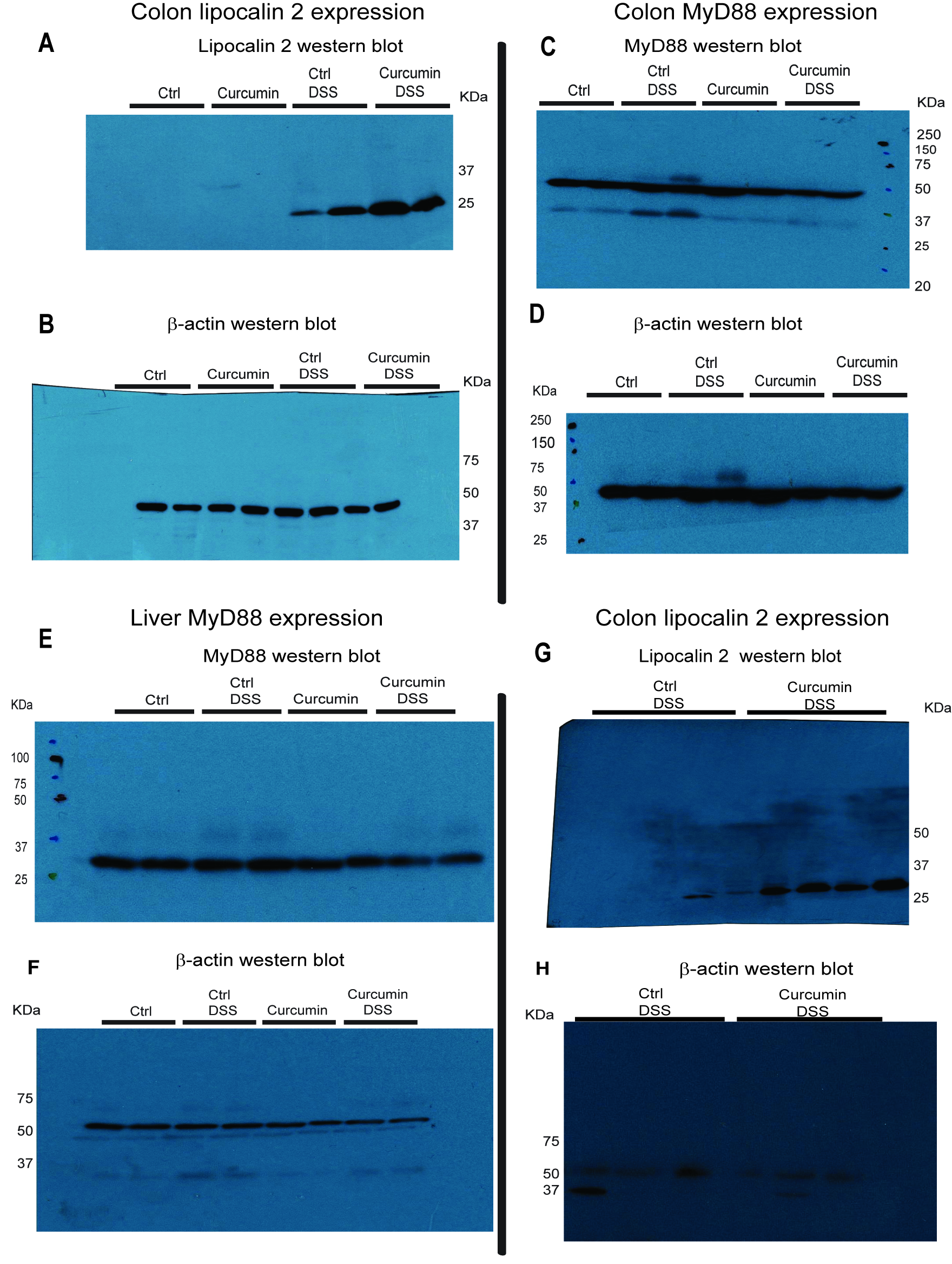

Supplement: S2 File — (A-B) Unaltered representative western blot of colon protein extracts of C57BL/6 mice probed with antibodies against lipocalin 2 (A) and (B) β-actin. Each lane represents an individual mouse. (C-D) Unaltered representative western blot of colon protein extracts of C57BL/6 mice probed with antibodies against (C) MyD88 and (D) β-actin. Each lane represents an individual mouse. (E-F) Unaltered representative western blot of liver protein extracts of C57BL/6 mice probed with antibodies against (E) MyD88 and (F) β-actin. Each lane represents an individual mouse. (G-H) Unaltered representative western blot of colon protein extracts of BALB/c mice probed with antibodies against (G) lipocalin 2 and (H) β-actin. Each lane represents an individual mouse. (TIF) [file pone.0208677.s002.tif]
